# Microglia Pyroptosis: A Candidate Target for Neurological Diseases Treatment

**DOI:** 10.3389/fnins.2022.922331

**Published:** 2022-07-22

**Authors:** Xian Wu, Teng Wan, Xiaoyu Gao, Mingyuan Fu, Yunfeng Duan, Xiangru Shen, Weiming Guo

**Affiliations:** ^1^Huazhong University of Science and Technology Union Shenzhen Hospital, The 6th Affiliated Hospital of Shenzhen University Health Science Center, Shenzhen, China; ^2^The First Affiliated Hospital of Hunan College of Traditional Chinese Medicine, Hunan Province Directly Affiliated TCM Hospital, Zhuzhou, China; ^3^Hengyang Medical College, University of South China, Hengyang, China

**Keywords:** microglia, pyroptosis, GSDMs, neuroinflammation, neurological diseases

## Abstract

In addition to its profound implications in the fight against cancer, pyroptosis have important role in the regulation of neuronal injury. Microglia are not only central members of the immune regulation of the central nervous system (CNS), but are also involved in the development and homeostatic maintenance of the nervous system. Under various pathological overstimulation, microglia pyroptosis contributes to the massive release of intracellular inflammatory mediators leading to neuroinflammation and ultimately to neuronal damages. In addition, microglia pyroptosis lead to further neurological damage by decreasing the ability to cleanse harmful substances. The pathogenic roles of microglia in a variety of CNS diseases such as neurodegenerative diseases, stroke, multiple sclerosis and depression, and many other neurological disorders have been gradually unveiled. In the context of different neurological disorders, inhibition of microglia pyroptosis by targeting NOD-like receptor family pyrin domain containing (NLRP) 3, caspase-1 and gasdermins (GSDMs) by various chemical agents as well as natural products significantly improve the symptoms or outcome in animal models. This study will provide new ideas for immunomodulatory treatment of CNS diseases.

## Introduction

The term “pyroptosis” is of ancient Greek origin, meaning fire and falling, it is characterized by early rupture of the plasma membrane and release of proinflammatory intracellular contents (Black et al., 1989). Therefore, pyroptosis is also known as inflammatory cell death in recent years (Van Opdenbosch and Lamkanfi, 2019). External stimuli such as pathogen-associated molecular patterns (PAMPs), damage-associated molecular patterns (DAMPs), dsDNA, multiple bacterial, viral antigenic components initiate cellular pyroptosis by activating different types of inflammasome receptors (Xue et al., 2019). Cell pyroptosis is mainly executed by the gasdermin family of proteins (Shi et al., 2017). The N-terminal end of the GSDM is activated by the shearing action of caspase family members. The activated GSDM-N terminus has membrane pore-forming properties and induces the formation of transmembrane pore channels and pyroptosis, along with the release of intracellularly activated inflammatory mediators (Bergsbaken et al., 2009; Shi et al., 2017). The GSDM plays an important role in the body's anti-infection and anti-cancer immunity and is expected to be a new target for anti-cancer therapy (Man et al., 2017; Loveless et al., 2021; Wu et al., 2021). However, cellular damage and inflammation caused by excessive and abnormal pyroptosis have been found to be important pathogenic factors in a variety of diseases, such as cardiovascular diseases, motor system diseases, autoimmune diseases (McKenzie et al., 2018; Tao et al., 2020; Wang et al., 2020c). Recent studies have shown that neuronal pyroptosis is associated with neurodegenerative diseases, stroke, traumatic brain injury (TBI), infection-induced brain damage, and a variety of central nervous system (CNS) pathologies such as epilepsy (McKenzie et al., 2020a). Pyroptosis has become the focus of research on the pathogenic mechanisms of the CNS.

Microglia are the resident innate immune cells of the CNS with important immunomodulatory functions and play a critical role in the neuronal development, myelin repair, and regeneration of the nervous system (Colonna and Butovsky, 2017; Lloyd and Miron, 2019). They can regulate inflammation and oxidative stress and influence synaptic plasticity and blood-brain barrier (BBB) stability, which are essential for the regulation of CNS homeostasis (Li and Barres, 2018; Zhou et al., 2019; Ronaldson and Davis, 2020). Although the concept of microglia polarization is currently controversial, the binary classification of microglia according to their beneficial or harmful function is still widely used today (Hu et al., 2015; Ransohoff, 2016a; Wan et al., 2022b). Recent studies suggest that its function is not limited to regulating development and simply removing cellular debris in disease, but that its abundance of modifiable genes also provides a large number of targets for regulating neurological diseases (Prinz et al., 2021). Given the multifunctional regulatory properties of microglia in the CNS, their association with epilepsy, neuropathic pain, ischemic stroke, neurodegenerative diseases, and depression are also being preliminarily revealed (Orihuela et al., 2016; Nestle et al., 2020; Yao et al., 2021). The links between the physiopathological functional state of microglia and various neurological diseases have been extensively investigated.

Microglia pyroptosis is an important manifestation of neuroinflammation and is closely associated with the development of several neurological diseases, such as ischemic brain injury, stroke, and neurodegenerative diseases. Microglia pyroptosis-induced inflammatory responses are associated with neurodegeneration and cell death in the brains of Parkinson's patients (Zhang et al., 2016). IL-1β and IL-18 levels were found to be significantly higher in the cerebrospinal fluid of Parkinson's patients with microglia pyroptosis than in the healthy controls (Zhang et al., 2016; Chang et al., 2020; Hu et al., 2020; Xu et al., 2021b). In addition, it was shown that inhibition of microglia pyroptosis by reducing the levels of the NLRP3 complex with CD73 or reduced advanced oxidation protein products (AOPPs) alleviated spinal cord injury (SCI) induced by persistent neuroinflammation (Liu et al., 2020; Xu et al., 2021b). In a mouse model of intracerebral hemorrhage (ICH), inhibiting microglia pyroptosis by suppressing NLRP3 complexes could improve SCI symptoms. In the ICH mouse model, the anti-inflammatory effect of Didymin was partially associated with its anti-microglia pyroptosis effect (Gu et al., 2022). This paper reviews the recent association between microglia pyroptosis and various neurological disorders, as well as the currently available or potential agents that regulate microglia pyroptosis, thus providing new ideas for the treatment of neurological disorders.

## Pyroptosis

### Pyroptosis as a Phenotype of Inflammatory Cell Death

Pyroptosis is a type of programmed cell death but is different from apoptosis and necrosis. Both pyroptosis and necrosis are part of the same lytic programmed death and eventually release contents leading to inflammation (Frank and Vince, 2019). However, the mechanisms and cell morphology are different, which may be due to the different biochemical activities of the gasdermin D (GSDMD) and mixed lineage kinase domain-like proteins (MLKL) that mediate pyroptosis (Jorgensen and Miao, 2015). Pyroptosis is one of the defense mechanisms of host cells against pathogens. Usually external pathogens promote cellular pyroptosis activation, while a few pathogens have the ability to inhibit host cell pyroptosis (Bergsbaken et al., 2009). With the rupture of the plasma membrane, exposed extracellular pathogens are further targeted and cleared by recruited immune cells. Classical microglia pyroptosis is divided into two main steps. The initiation step refers mainly to microbial and inflammatory mediator-mediated activation of inflammation-associated genes. This is followed by the activation of inflammasome and downstream pyroptosis-related pathways, culminating in cell death mediated by the pore-forming protein GSDMD (Vande Walle and Lamkanfi, 2016; Fang et al., 2020). In addition, the non-classical pyroptosis pathway GSDMD activation is independent of caspase-1. Caspase-4/5/11 are directly stimulated by LPS and thus activate downstream GSDMD (Shi et al., 2014).

### Mechanism and Regulation of Pyroptosis

Inflammasomes are multiprotein complexes containing pattern recognition receptors (PRRs) that mediate the innate immune response of the body to infectious microbes and host protein molecules. The PRR family typically contains multiple members, including Toll-like receptors (TLRs), Nod-like receptors (NLRs) (Kanneganti et al., 2007; Sahoo, 2020). PRRs in inflammasomes sense and recognize PAMPs and DAMPs of host or environmental origin (Guo et al., 2015). In mammals, in addition to the most common NLRP3, different types of inflammasome receptors such as absent in melanoma 2 (AIM2), NLRC4, NLRP1b, and NLRP6 recognize different activation signals and activate downstream caspase-1 (Xue et al., 2019; Fang et al., 2020). Upon activation, caspase-1 mediates the shear activation of IL-1β and IL-18, as well as GSDMD, leading to the formation of membrane pores, cell swelling, release of cell contents, and ultimately pyroptosis (Voet et al., 2019; Wang et al., 2019; Xue et al., 2019). Because NLRP3 inflammasomes have a wide recognition range, targeting NLRP3 to regulate pyroptosis has become one of the focal points of current research. Isoflurane general anesthesia induces pyroptosis by activating NLRP3 inflammasomes, while NLRP3 inhibitor MCC950 attenuates pyroptosis-related cognitive dysfunction (Fan et al., 2018). Additionally, isoliquiritin and kanglexin improve depression by downregulating NLRP3 levels and subsequent neuronal pyroptosis (Bian et al., 2020; Li et al., 2021a).

GSDMD is highly expressed in the epithelium and skin of the gastrointestinal tract, yet its function remains largely unknown. As the executioner of pyroptosis, GSDMD is only one member of the GSDM family, with other members including GSDMA, GSDMB, GSDMC, GSDMD, DFNA5, and DFNB59 (Shi et al., 2017). In the GSDM family, the sequence homology is about 45%, with the GSDM-N structural domain being the most conserved region. It was concluded that the GSDM-N domain of GSDMD is thought to possess extremely strong pore-forming toxicity and then induce pyroptosis (Shi et al., 2015; Ding et al., 2016). In studies in mouse models, different species of GSDM activation have similar pore-forming properties. At present, the upstream and downstream signal regulation mechanism of pyroptosis executive protein GSDMD is clearly studied (Shi et al., 2017). Recent studies have found that streptococcal pyrogenic exotoxin B shears GSDMA and thus induce pyroptosis, while the upstream regulatory mechanisms of the remaining GSDM members are still unknown (Deng et al., 2022). The GSDMD proteins are in an autoinhibited state in the normal state (Ding et al., 2016). GSDMD contains about 480 amino acids, and it is connected to two structural domains, GSDM-N terminus and GSDM-C terminus, by a long loop. Activated caspase-1 and caspase-11 efficiently cleave the GSDMD at an aspartate site within the loop. This cleavage is essential for release of pore-forming GSDM-N terminus (Shi et al., 2015). The role of pyroptosis in CNS disease has emerged in multiple animal disease models. VX-765, a small molecule inhibitor of caspase-1, reduces the expression of inflammasomes and pyroptosis-associated proteins in the CNS, thereby inhibiting axonal injury in multiple sclerosis (MS) (McKenzie et al., 2018). Caspase-1 gene ablation in mice model significantly inhibited TBI-induced pyroptosis and neurological damage (Liu et al., 2018).

Studies on the molecular regulation of its non-canonical pathway of pyroptosis, especially caspase-11-related, are currently more limited. LPS-mediated caspase-11-dependent non-classical pyroptosis pathway can be inhibited by ethyl pyruvate (Qiu et al., 2020). Regulation of caspase-11 is thought to be possibly related to phosphodiesterase 8A (PDE8A) mediated cyclic adenosine monophosphate (cAMP) metabolism (Hou et al., 2019). Adenosine diphosphate (ADP) -ribosylation of caspase-11 was found to protect cells from pyroptosis in Shigella-infected mice (Li et al., 2021b). AIM2 is also a target for regulating pyroptosis. *In vitro*, andrographolide significantly inhibited AIM2 inflammasomes and blocked caspase1/GSDMD-mediated myeloid-derived macrophage pyroptosis (Gao et al., 2019). LncRNA MEG3 induced cellular pyroptosis of middle cerebral artery occlusion (MCAO) mice in ischemic brain by sponging miR-485 targeting AIM2 (Liang et al., 2020a). Thus, non-coding RNAs are also important for regulating pyroptosis. In cellular experiments in diabetic retinopathy, overexpression of miR-590-3p was found to downregulate caspase-1-dependent pyroptosis *via* downregulation of NLRP1 and downstream NADPH oxidase 4 (NOX4) pathway (Gu et al., 2019). As the study of pyroptosis in human diseases has advanced, chemotherapeutic drugs and miRNAs have now been found to inhibit malignant progression of tumors by inducing tumor pyroptosis (Xia et al., 2019). Targeting pyroptosis has an unignorable role in the treatment of diseases.

### Crosstalk Between Pyroptosis and Other Kinds of Cell Death

There is a link between apoptosis and pyroptosis. After apoptosis, the absence of macrophages to remove the apoptotic cells trigger GSDMD-mediated secondary cell death with a pathological pattern similar to pyroptosis (Kovacs and Miao, 2017; Rogers et al., 2017). When GSDMD expression is too low or in the presence of GSDMD defects, caspase-1 induces apoptosis *via* the Bid-caspase 9-caspase 3 axis or caspase-7 (Taabazuing et al., 2017; Tsuchiya et al., 2019). However, if the cleavage site of GSDMD is designed as a recognition site for caspase-3, it may convert apoptosis into pyroptosis (Wang et al., 2017). In addition, oxidation of phospholipids may also be associated with GSDMD activation-induced pyroptosis. Glutathione peroxidase 4 (GPX4) and vitamin E inhibit pyroptosis in mouse macrophages by suppressing lipid peroxidation (Imai and Nakagawa, 2003). In contrast, myeloid-specific GPX4 deficiency leads to a significant increase in caspase-1 and caspase-11-mediated GSDMD lysis (Kang et al., 2018). Meanwhile, GPX4 plays an important role in regulating ferroptosis. This suggests the potential connection between pyroptosis and ferroptosis.

## Microglia Pyroptosis and Neurological Diseases

### Neurodegenerative Diseases

Neurodegenerative diseases are a series of disorders caused by progressive loss of neurons in the CNS (Yu et al., 2017). The main pathological changes include amyloid deposition and progressive neurodegenerative changes. Among them, amyloid proteins including Aβ, tau and α-synuclein exhibit similar properties to prion proteins in pathological experiments and are able to self-replicate and spread throughout the nervous system (Vaquer-Alicea and Diamond, 2019; Tian et al., 2020). Microglia play an important role in the course of neurodegenerative diseases (Wan et al., 2022a). In the development of Alzheimer's disease (AD), the caspase activation and recruitment domain (CARD/ASC)-containing bridging proteins released by microglia pyroptosis rapidly bind to Aβ and increase the formation of Aβ oligomers and aggregates (Venegas et al., 2017). The formed ASC-Aβ complex also leads to multiple responses in surrounding cells, such as increased caspase-1 activation, IL-1β maturation and GSDMD cleavage, and promotes NLRP3 inflammasome formation and pyroptosis in neighboring microglia (Heneka et al., 2018; Luciunaite et al., 2020). In addition, microglia pyroptosis lead to more ASC release, thereby exacerbating pyroptosis-induced neuroinflammatory damage (Friker et al., 2020). The ASC-Aβ complex also promotes microglia activation and secretion of inflammatory mediators and neurotoxic cytokines, while the efficiency of Aβ degradation in microglia is reduced (Sarlus and Heneka, 2017). In the early stages of AD, microglia in patients are activated by Aβ, which is removed by receptor-mediated phagocytosis and degradation, inhibiting to some extent the deposition of Aβ in the interstitial (Newcombe et al., 2018). However, as Aβ accumulates, microglia are continuously activated and produce excessive amounts of pro-inflammatory cytokines (Van Zeller et al., 2021). In addition, the autocrine secretion of membrane receptors from microglia that bind and help microglia phagocytose Aβ gradually decreases, and the activity of various degradative enzymes, such as enkephalinase, insulin-degrading enzymes, and angiotensin-converting enzyme I (ACEI) decreases, further leading to reduced clearance of Aβ and continued microglial cell stimulation (Yu and Ye, 2015; Van Zeller et al., 2021). Researchers have speculated that tau and Aβ are functionally similar. Tau protein also activates inflammasomes and induces microglia pyroptosis *via* the NLRP3-ASC axis (Stancu et al., 2019). This finding is supported by the results of several necropsy analyses (Ransohoff, 2016b; Leyns and Holtzman, 2017).

The pathological marker of Parkinson's disease (PD) differs considerably from that of AD. The pathological marker of PD is fibrillar α-synuclein, which tends to accumulate in neurons and eventually leads to the formation of Lewy bodies (Przedborski, 2017). Similar to Aβ, α-synuclein also induces the activation of microglia NLRP3 inflammasomes and thus promotes the release of ASC from microglia and the formation of extracellular ASC patches (Zhou et al., 2016; de Alba, 2019). Delayed and strong activation of NLRP3 inflammasomes and a significant increase in extracellular ASC release were observed in LPS and α-synuclein-stimulated activated mouse microglia, but microglia did not undergo pyroptosis (Gordon et al., 2018). Furthermore, activation of pyroptosis-related pathways was associated with the metabolism of α-synuclein. It was shown that the activation and aggregation of inflammasomes in neuronal cells are closely related to the cleavage of extracellular α-synuclein (Wang et al., 2016; Hu et al., 2022). In BE (2)-M17 human dopaminergic neuroblastoma cells, caspase-1 activated by inflammasomes was found to cleave α-synuclein *in vitro* and produce aggregates with neuronal toxicity (Wang et al., 2016). In a rat model of PD induced by LPS and 6-hydroxydopamine (6-OHDA), NLRP3 inflammasomes components were found to be highly expressed in microglia, and caspase-1 inhibitor (Ac-YVAD-CMK) reversed this result (Mao et al., 2017). This suggests that microglia pyroptosis may be associated with pathological cleavage of extracellular α-synuclein and the formation of Lewy bodies. However, whether α-synuclein ultimately induces microglia pyroptosis may depend on the concentration of α-synuclein, and the quantification of this concentration needs to be determined by further studies. Furthermore, in N-methyl-4-phenyl-1,2,3,6-tetrahydropyridine (MPTP)-induced PD mice, baicalein inhibited NLRP3/caspase-1/GSDMD pathway-mediated microglia pyroptosis, thereby reducing PD symptoms (Rui et al., 2020). These studies suggest that inhibition of microglia pyroptosis may alleviate the progression of PD.

Caspase-1 plays a key role in the process of pyroptosis. The activation of Caspase-1 exists in the brain of Huntington's disease (HD) patients and in HD mouse models, and the inhibition of caspase-1 in HD mouse models can slow down the progression of the disease (Ona et al., 1999; Paldino et al., 2020). Huntington's protein (HTT) is the key to the disease and activated caspase-1 hydrolyzes and cleaves HTT to produce an N-terminal mutated fragment (N-htt), leading to neuronal dysfunction and death (Kim et al., 2001; Sanchez Mejia and Friedlander, 2001). This suggests that pyroptosis may be potentially linked to the pathological generation of HTT. The current study shows that HD patients have distinct neuroinflammatory features at the site of brain lesions, while no upregulation of immune cells from the periphery, such as lymphocytes and neutrophils, was found in the brain tissue of HD patients (Bjorkqvist et al., 2008; Palpagama et al., 2019). Significant NLRP3 activation was found in microglia in a mouse model of HD, suggesting a potential link between neuroinflammation triggered by microglia pyroptosis and HD (Siew et al., 2019). However, a different result has been obtained that NLRP3 expression levels were significantly elevated in other cells of the striatum of HD mice, but no significant NLRP3 activation was found in microglia (Paldino et al., 2020). This heterogeneity of results due to the spatial location of the brain needs to be elucidated by more in-depth studies.

### Ischemic Stroke

Ischemic stroke and secondary cerebral ischemia-reperfusion injury are both very serious cerebrovascular diseases (Graeser et al., 2019). Ischemia and hypoxia trigger a series of neurological damage responses such as oxidative stress and neuroinflammation (Langhauser et al., 2012; Nabavi et al., 2015). Neuroinflammation induced by microglia pyroptosis is thought to be a key factor promoting neuronal damage after ischemia (Ceulemans et al., 2010). On the one hand, neuroinflammation promotes the clearance of dead cellular debris induced by reduced cerebral blood flow and ischemia-reperfusion (Xu et al., 2019a). On the other hand, it may lead to infarct exacerbation and low neuronal plasticity (Kriz, 2006). Microglia are one of the most important phagocytic cells for the removal of necrotic substances, but hyperactivation-induced microglia pyroptosis is an important cause of exacerbation of neuroinflammation-related damage after stroke (Iadecola and Anrather, 2011; Xu et al., 2019a). In the mouse MCAO-induced I/R model, microglia GSDMD expression is elevated in the ischemic region, which mediates microglia pyroptosis and neuroinflammation-related injury (Voet et al., 2019; Zhang et al., 2019; Wang et al., 2020b).

It has been found that NLRP3 expression is increased in microglia of ischemic stroke patients, and increased expression of NLRP3 inflammasomes component proteins and downstream products IL-1β and IL-18 was also observed in the mouse MCAO/R model (Fann et al., 2013). Many studies have revealed that the mechanism of NLRP3 inflammasome in microglia involved in the regulation of cerebral ischemic injury may be related to the NF-κB pathway, mitogen-activated protein kinase (MAPK) signaling pathway, Hypoxia Inducible Factor-1α (HIF-1α), reactive oxygen species (ROS) production (Ma et al., 2014; Fann et al., 2018; Jiang et al., 2020). In oxygen-glucose deprivation and reoxygenation (OGD/R) and MCAO/R-treated rat BV2 microglia, Salidroside (Sal) was found to inhibit microglia NLRP3 inflammasomes activation by suppressing the TLR4/NF-κB signaling pathway, thereby inhibited I/R-induced BV2 cells pyroptosis and further neuronal damage (Liu et al., 2021). A similar phenomenon was observed from the Meisoindigo-treated MCAO mouse model (Ye et al., 2019; Liu et al., 2021). These studies suggest that amelioration of ischemic stroke can be achieved by inhibiting the TLR4/NF-κB signaling pathway. In a human cell assay, ROS levels in OGD/R-treated human BV2 microglia were significantly higher than those in the untreated group, and co-culture with hypoxia-pretreated olfactory mucosa mesenchymal stem cells (OM-MSCs) showed reduced ROS levels and less pyroptosis (Huang et al., 2020). OM-MSCs have immunomodulatory and reparative functions and replace or repair damaged cells, and OM-MSCs upregulate the expression and release of HIF-1α under OGD/R conditions, suppressing the expression of NLRP3 inflammasome and pyroptosis-related proteins in co-cultured BV2 microglia and reducing the pyroptosis of BV2 microglia under OGD/R conditions (Coppin et al., 2019; Dabrowska et al., 2019; Huang et al., 2020). These studies suggest that ischemia-induced production of HIF-1α and ROS play a key role in pyroptosis regulation in BV2 microglia under OGD/R conditions. Another study found that NLRP3 inflammasomes expression levels were not altered in a mouse OGD model, and only NLRC4 inflammasome expression was upregulated and induced BV2 microglia pyroptosis. Knockdown of NLRC4 by siRNA significantly reduced pyroptosis in microglia under ischemic conditions (Poh et al., 2019). The difference in NLRP3 and NLRC4 expression under ischemic stroke conditions remains to be elucidated.

### Multiple Sclerosis

MS is an incurable progressive demyelinating disease of the CNS characterized by multiple demyelinating plaques in the white matter, neurodegeneration, and axonal transection or loss (Kornek and Lassmann, 2003; Dendrou et al., 2015). The etiology of MS is not yet clear. Previous studies have shown increased expression of NLRP3 inflammasomes activation and its downstream products in CNS tissue and peripheral serum in MS patients compared to non-MS patients, leading to BBB damage and neurotoxicity (Huang et al., 2004; Burm et al., 2016; McKenzie et al., 2018). In addition, a study found a large number of GSDMD-immunopositive cell fragments in the frontal white matter of cadavers from MS patients, suggesting a potential link between GSDMD-mediated microglia pyroptosis and MS (McKenzie et al., 2018). Because MS is a human-specific disease, it can only be partially simulated in animal models, such as the animal experimental autoimmune encephalomyelitis (EAE) model, which has approximately the same neuropathological features as MS and assist in determining the factors influencing MS (Ransohoff, 2012; Kipp et al., 2017). It has been found that the expression level of NLRP3/ASC-caspase/GSDMD pathway is significantly increased in mouse EAE models, while the use of Liraglutide significantly downregulates the protein level of caspase-1 and microglia pyroptosis levels (Song et al., 2022). Another study showed that in addition to caspase-1, caspase-3 and caspase-7 also mediated microglia pyroptosis in post-mortem brain tissue from patients with progressive MS and in a mouse EAE model. This study found that caspase-1 activates caspase-3/7 in MS patients and EAE mouse models, and caspase-3/7 and its substrates, such as PARP, DFF45 and ROCK1, after cleavage activation, induce GSDMD-mediated microglial cell pyroptosis by disrupting the cellular protein hydrolysis network and promoting microglial cell nuclear cohesion glial cell pyroptosis (McKenzie et al., 2020b).

### Major Depressive Disorder

**Figure 1:**
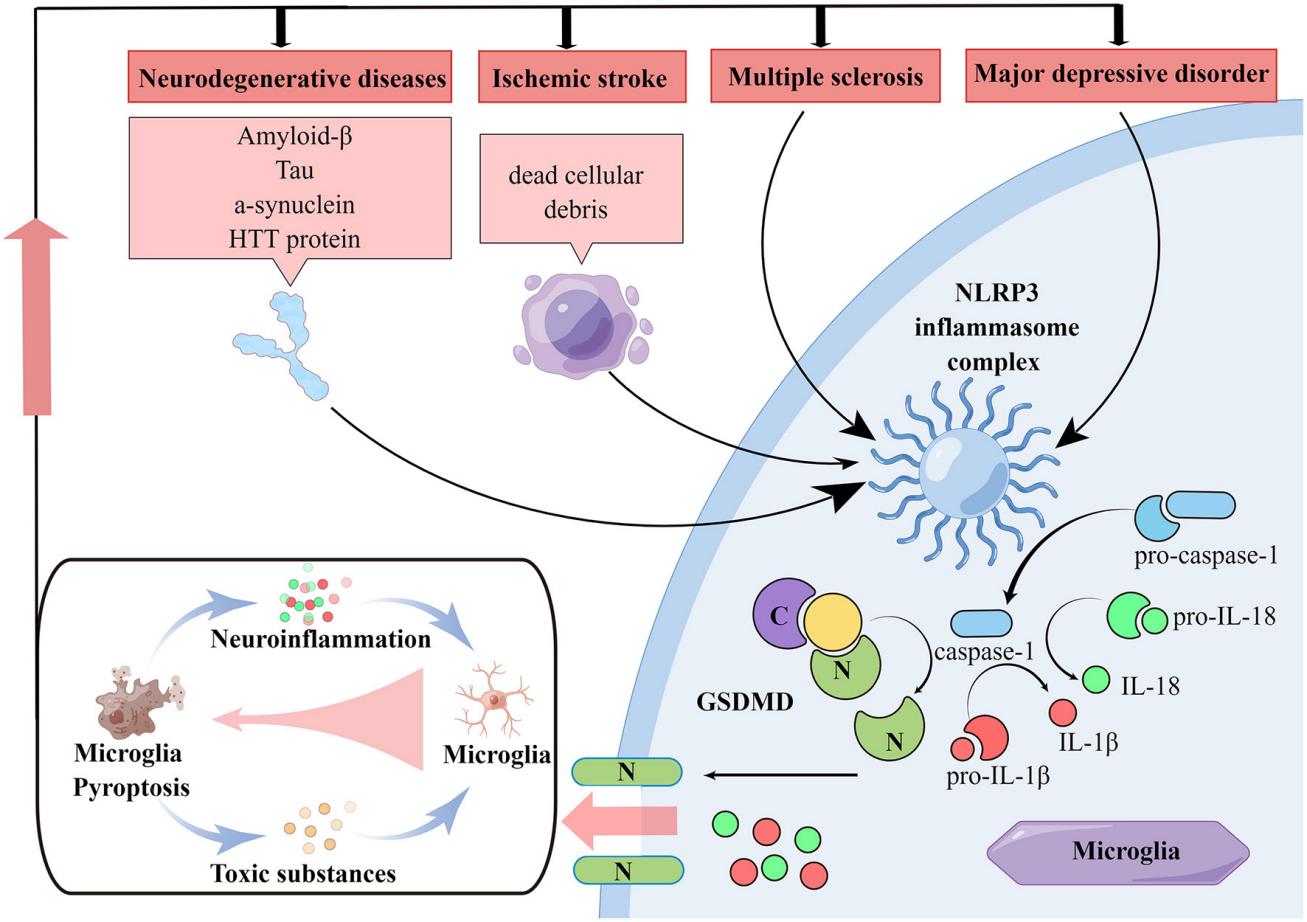
Pathologies of neurological diseases associated with microglia pyroptosis. In various neurological diseases, NLRP3 inflammasome in microglia can be activated and then the caspase-1 is activated. Activated caspase-1 on the one hand cleaves GSDMD, which produces N-terminal structural domain and induces cell membrane perforation and subsequent pyroptosis. On the other hand, activated caspase-1 cleaves IL-1β and IL-18 precursors, rising the extracellular level of IL-1β and IL-18 and amplify neuroinflammatory responses. In neurodegenerative diseases, various misfolded aggregated proteins are taken up by microglia, leading to NLRP3 inflammasome activation and microglia pyroptosis. While in ischemia stroke, ischemic necrotic cell debris is taken up by microglia performing immune clearance functions, leading to microglia NLRP3 inflammasome activation. Microglia NLRP3 inflammasome activation is also present in MDD and MS. All these diseases lead to NLRP3-dependent microglia pyroptosis and neuroinflammation. Moreover, worsening neuroinflammation promotes the production of multiple pathological markers of neurological disease and induces microglia pyroptosis, which leads to persistent disease progression.

Major depressive disorder (MDD) is a serious neuropsychiatric disorder that remains a medical management challenge (Malhi and Mann, 2018). The direct pathogenesis of depression remains unclear, and some studies now suggest a close relationship between MDD and neuroinflammation triggered by NLRP3 inflammasomes (Raedler, 2011; Dey and Hankey Giblin, 2018). In patients with MDD, untreated patients have increased levels of IL-1β and IL-18 in the circulation and increased expression of NLRP3 compared to patients treated with the antidepressant amitriptyline (Raedler, 2011). The expression of NLRP3 was increased. Elevated IL-1β mRNA and protein levels were also found in the prefrontal cortex in a chronic mild stress (CMS)-induced depression model in rats, while no similar phenomenon was observed in blood (Pan et al., 2014). The same phenomenon was not observed in blood. In the chronic unpredictable mild stress (CUMS)-induced depression mouse model, researchers found significantly elevated levels of IL-1β protein in serum and hippocampus, but not in NLRP3 knockout mice, probably because NLRP3 knockout inhibited MAPK pathway and NF-κB pathway activation (Su et al., 2017). Many studies have also revealed other mechanisms involved in the regulation of MDD by NLRP3 inflammasomes in microglia, which may be related to dysregulation of miRNA-27a/SYK/NF-κB pathway, nuclear factor-erythroid 2 -related factor 2 (Nrf2) (Ajami et al., 2011; Eggen et al., 2013; Arioz et al., 2019; Li et al., 2021a). In the LPS-induced mouse Depressive-like behavior (DLB) model, Melatonin pretreatment inhibited Keap-1-mediated Nrf2 proteasomal degradation, suppressed NLRP3 inflammasomes activation, downregulated GSDMD cleavage, and ultimately protected N9 microglia from pyroptosis (Arioz et al., 2019). Similarly, in MDD patients and in LPS or chronic social defeat stress (CSDS)-induced depression models in mice, the use of Isoliquiritin upregulated miRNA-27a expression and downregulated SYK expression, thereby protecting microglia from pyroptosis and alleviating MDD symptoms in mice (Li et al., 2021a). These experiments illustrate that microglia pyroptosis is closely linked to MDD. Interestingly, in a CMS-induced mouse depression model, astrocyte NLRP3 inflammasomes and GSDMD in the hippocampus of mice were found to activate and induce cell pyroptosis, while microglia did not show significant pyroptosis (Catanese et al., 2021) (Figure 1).

## Regulation of Microglia Pyroptosis for Treatment of Neurological Diseases

### Targeting the Regulation of NLRP3

Targeting NLRP-related proteins to regulate microglia pyroptosis affect the progression of multiple neurological diseases. Targeting NLRP to inhibit pyroptosis plays a neuroprotective role in stroke. The human-specific gene CHRFAM7A inhibits NLRP3 inflammasomes activation and reduces intracellular levels of NLRP3, a pyroptosis-related protein, thereby reducing OGD/R-induced neurological damage (Cao et al., 2021). The triggering receptor expressed on myeloid cells (TREM)-1 antagonist LP17 inhibited microglia pyroptosis and effectively improved neurological function in subarachnoid hemorrhage (SAH) patients (Liang et al., 2020b; Xu et al., 2021a). C-C chemokine receptor 5 (CCR5) activation promotes microglia pyroptosis and neurological deficits after ICH in mice *via* the protein kinase A (PKA)/cAMP response element binding (CREB)/NLRP1 signaling pathway. Maraviroc inhibition of CCR5 improves neurological function in ICH patients (Yan et al., 2021). Dexmedetomidine (Dex) inhibits microglia pyroptosis by blocking the purinergic 2X7 receptor (P2X7R)/NLRP3 pathway, thereby providing protection against ischemic brain injury (Sun et al., 2021). Andrographolide and Curcumin effectively reduce neurostructural damage and functional impairment caused by stroke by inhibiting NF-κB signaling and NLRP3 inflammasomes-mediated microglia pyroptosis (Li et al., 2018; Ran et al., 2021). Salvianolic acids for injection (SAFI) reduced brain ischemia reperfusion injury (BIRI) by reducing the shift in microglia phenotype from M1 to M2 and inhibiting microglia NLRP3 inflammasomes-induced pyroptosis (Ma et al., 2021). The plasma containing Melatonin has been shown to reduce BIRI. Melatonin-containing plasma exosomes inhibited ischemia-induced inflammatory responses and pyroptosis by modulating the TLR4/NF-κB signaling pathway (Wang et al., 2020a).

Targeting NLRP3 to inhibit microglia pyroptosis inhibition alleviates cognitive impairment. NLRP3 inhibitors MCC950 and the ethyl acetate fraction of Bungeanum improve cognitive function in mice by inhibiting LPS-induced caspase-1 activation and pyroptosis in microglia (Dempsey et al., 2017; Zhao et al., 2021). Curcumin treatment significantly improved diabetes mellitus (DM)/chronic cerebral hypoperfusion (CCH)-induced cognitive impairment by modulating the TREM2/TLR4/NF-κB pathway and reducing NLRP3-dependent pyroptosis (Zheng et al., 2021). Studies have shown that chronic aluminum exposure is associated with the development of AD and cognitive impairment (Klotz et al., 2017). Aluminum exposure causes microglia pyroptosis and neuroinflammation through the DEAD-box helicase 3 X-linked (DDX3X)/NLRP3 inflammasomes signaling pathway, while Resveratrol alleviates aluminum exposure-induced neurological damage by activating sirtuin1 (SIRT1) (Hao et al., 2021). Hypoxic preconditioning of the Ca2+/calcium/calmodulin-dependent protein kinase II (CaMKII)/CREB signaling pathway inhibited microglia pyroptosis and thereby improved Amyloid precursor protein (APP)/CREB signaling in presenilin-1 (PS1) mice with brain damage (Song et al., 2021).

A variety of agents effectively inhibit PD progression by targeting microglia pyroptosis. Baicalein reverses MPTP-induced neuroinflammation in mice by inhibiting the NLRP3/caspase-1/GSDMD pathway and has a role in PD treatment (Rui et al., 2020). Kaemperfol ameliorates behavioral deficits in PD rats by inhibiting p38MAPK/NF-κB pathway, inhibiting microglia activation, and downregulating pyroptosis-related proteins (Cai et al., 2022). In a mouse model of depression, Quercetin (Qu) and Isoliquiritin treatment inhibited microglia pyroptosis-mediated neurotoxicity and thus exerted antidepressant effects (Han et al., 2021; Li et al., 2021a).

Notably, nuclear factor-kappa B (NF-κB) signaling plays a key role in the formation of NLRP3 inflammasomes in the study of the whole range of pyroptosis regulation-related signals (Yuan et al., 2021). Para-aminosalicylic acid (PAS-Na) antagonizes Mn-induced activation of NLRP3 inflammasomes in the basal ganglia of rats by inhibiting activation of the NF-κB pathway and oxidative stress induced by BV2 cell pyroptosis (Peng et al., 2020). Nrf-2 activates heme oxygenase-1 (HO-1), and Dimethyl itaconate (DI) are involved in the Nrf-2/ HO-1 pathway inhibit NLRP3 inflammasomes assembly and GSDMD cleavage and induces cellular autophagy (Yang et al., 2021). Sulforaphane (SFN) and Dimethyl fumarate (DMF) activate nuclear factor erythroid 2-related factor 2 (Nrf2) and inhibit NF-κB, thereby inhibiting NLRP3 inflammasomes formation and subsequent microglial cell pyroptosis in mice (Tastan et al., 2021; Tufekci et al., 2021). In palmitic acid-treated BV2 cells, miR-124 inhibited microglia pro-inflammatory responses by suppressing the TLR4/myeloid differentiation factor 88 (MyD88)/NF-κB signaling pathway (Yang et al., 2022). Microglia pyroptosis plays a crucial role in secondary injury of SCI (Xu et al., 2020). Celastrol inhibits microglia activation and NF-κB/p-p65 expression *in vivo* and *in vitro*, and attenuates the inflammatory response in SCI induction (Dai et al., 2019).

### Regulation of Caspase and GSDM

Given the important role of caspases and GSDM family-related proteins in cell pyroptosis, numerous drug trials targeting them have been initiated. AC-YVAD-CMK, a selective inhibitor of caspase-1, was found to inhibit microglia pyroptosis and induce an anti-inflammatory phenotype in microglia, thereby improving ICH mice (Lin et al., 2018). The caspase-1 inhibitor VX765 has been found to reduce neurological damage after TBI by inhibiting pyroptosis and the high-mobility cassette-1/TLR4/ NF-κB pathway activity, resulting in a better therapeutic effect on TBI (Sun et al., 2020). Clinical doses of sevoflurane exacerbated AD progression *via* the NLRP3/caspase-1/GSDMD axis. VX-765 significantly inhibited the activation of microglia pyroptosis-related pathways and attenuated sevoflurane-induced release of IL-1, IL-18 and tau-related kinases and phosphatases (Xu et al., 2019b; Tian et al., 2021a). Paeoniflorin (PF) exerted antidepressant effects by inhibiting caspase-11-dependent pyroptosis signaling induced by hyperactivation of hippocampal microglia in ricin-treated mice, and attenuated neuroinflammatory responses (Tian et al., 2021b). Under neuroinflammatory conditions, caspase-3/7 activation promotes GSDMD-associated microglia pyroptosis, and inhibition of GSDMD by siRNA transduction inhibits microglia pyroptosis, providing a new therapeutic opportunity for neuroinflammatory diseases such as MS (McKenzie et al., 2018, 2020b).

Mafenide (MAF) inhibits GSDMD cleavage through direct binding to the GSDMD-Asp275 site, downregulates p30-GSDMD expression, and suppresses bone marrow-derived macrophages (iBMDM) and BV2 microglia pyroptosis (Han et al., 2020b). In contrast, Sulfa-4 and Sulfa-22 target GSDMD cleavage, inhibit pyroptosis and inflammatory factor release, and have a therapeutic effect on neuroinflammation in AD (Esmaeili-Mahani et al., 2021). MiRNA-22 was negatively correlated with the expression of inflammatory factors in AD patients (Han et al., 2020a). Adipose-derived mesenchymal stem cells miRNA-22 loaded exosomes (Exo-miRNA-22) inhibited microglia pyroptosis and decreased inflammatory factor release by targeting GSDMD, and improved neurological function in AD mice (Zhai et al., 2021). GSDMD-mediated microglia pyroptosis is involved in kainic acid-induced seizures, and DMF, as an inhibitor of GSDMD N-terminal fragments (GSDMD-N), significantly reduce microglia pyroptosis and the expression of inflammatory factors such as IL-1 and IL-18, and play a certain role in the treatment of epilepsy (Xia et al., 2021). In SCI, the immunosuppressive molecule CD73 attenuates GSDMD-mediated microglia pyroptosis by promoting the phosphatidylinositol 3-kinase (PI3K)/AKT/Foxo1 signaling pathway (Xu et al., 2021b). Table 1 graphically covers the mechanisms in which the various drugs mentioned above modulate neurological disorders by targeting microglia pyroptosis.

**Table 1:** Regulation of microglia pyroptosis by various reagents for treatment of neurological diseases.

**Reagent**	**Objectives**	**Significance**	**Mechanism**	**References**
OGD/R	Cerebral I/R injury patients	Attenuated cerebral I/R injury	Inhibiting microglia pyroptosis in a NLRP3/Caspase-1 pathway-dependent manner and promoting microglia polarization to M2 phenotype	Eggen et al., 2013
LP17	SAH mouse model	Ameliorated microglial pyroptosis	Diminishing levels of GSDMD-N and IL-1β production	Catanese et al., 2021
LP17	MCAO rat model	Ameliorated neuronal damage and alleviates neuro-inflammation	Reducing oxidative stress and pyroptosis	Cao et al., 2021
MVC	Adult male ICH mice	Ameliorated neuronal pyroptosis and neurological deficits	Inhibiting CCR5/PKA/CREB/NLRP1 signaling pathway	Xu et al., 2021a
Dex	p-MCAO rat model	Inhibited microglia pyroptosis	Blocking the P2X7R/NLRP3/Caspase-1 pathway	Liang et al., 2020b
Andro	Adult SBI male rats	Inhibited microglia pyroptosis and reduced neuronal cell death and degeneration	Inhibiting NF-κB signaling pathway and suppressing the assembly of NLRP3 inflammasome	Yan et al., 2021
curcumin	MCAO mice model	Attenuated microglial pyroptosis	Suppressing NF-κB/NLRP3 signaling pathway	Sun et al., 2021
SAFI	MCAO/R rat model and OGD/R co-cultured primary neurons and primary microglia model	Exert neuroprotective effect	Reducing neuronal apoptosis, switching microglial phenotype from M1 toward M2, and inhibiting NLRP3 inflammasome/ pyroptosis axis in microglia	Li et al., 2018
Basal plasma exosomes melatonin	Focal cerebral ischemia rat model	Decreased neuroinflammation and microglial pyroptosis	Regulation of the TLR4/NF-κB signaling pathway	Ran et al., 2021
MCC950	APP/PS1 mouse model	Reduced Aβ accumulation and improved cognitive function	Inhibiting caspase 1, inflammasome and microglial activation	Ma et al., 2021
Z. bungeanum	Aging mice model and LPS/ATP-induced BV-2 microglial cells	Ameliorated cognitive deficits	Ameliorating oxidative stress and suppressing the NLRP3 inflammasome pathway and GSDMD-mediated pyroptosis	Wang et al., 2020a
Curcumin	DM and CCH rat model	Improved DM/CCH-induced cognitive deficits and attenuated neuronal cell death	Suppressing neuroinflammation induced by microglial activation, regulating the TREM2/TLR4 /NF-κB pathway, alleviating apoptosis and reducing NLRP3-dependent pyroptosis	Dempsey et al., 2017
Rsv	AlCl3 mice model	Ameliorated neuroinflammation and cognitive deficits	Activating SIRT1	Zheng et al., 2021
U50488H	APP/PS1 mouse model	Inhibited microglia pyroptosis and improved the synaptic plasticity	Inhibiting the Ca 2+/CaMKII/CREB signaling pathway	Klotz et al., 2017
MPTP	PD mice model	Reversed MPTP-induced neuroinflammation	Suppressing NLRP3/caspase-1/GSDMD pathway	Hao et al., 2021
6-OHDA	PD rat model and BV2 inflammatory cells	Inhibited microglia pyroptosis and neuroinflammatory response	Inhibiting p38MAPK/NF-κB signaling pathway	Song et al., 2021
Isoliquiritin	Depressed patients and mice	Decreased microglia pyroptosis	Inhibiting miRNA-27a/SYK/NF-κB signaling pathway	Deng et al., 2022
Qu	Depression and PD mouse models	Ameliorated neuronal injury	Inhibiting mtROS-mediated NLRP3 inflammasome activation	Rui et al., 2020
Mn	BV2 microglial cell line and male rats	Inhibited NLRP3 inflammasome dependent pyroptosis	Inhibiting NF-κB pathway activation and oxidative stress	Han et al., 2021
DI	BV2 microglial cells line	Inhibited microglia pyroptosis	Regulating Nrf-2/HO-1 pathway	Yuan et al., 2021
SFN	Murine microglial cells	Suppressing NLRP3 inflammasome and microglia pyroptosis	Inhibiting NF-κB nuclear translocation and Nrf2 mediated miRNAs expression modulation	Peng et al., 2020
DMF	N9 microglial cells	Inhibiting pyroptotic cell death	Decreasing miR-146a and miR-155 and regulating Nrf-2/HO-1 pathway	Yang et al., 2021
Palmitic acid	BV2 cells	Preventing microglial proinflammatory response	Downregulating TLR4/MyD88/NF-κB p65 signaling	Tufekci et al., 2021
-	SCI BV2 cells	Enhancing microglial pyroptosis	Activating PI3K/AKT pathway and promoting the expression of lncRNA-F630028O10Rik	Tastan et al., 2021
Celastrol	SCI rat model	Attenuated inflammatory response	Inhibiting the expression of NF-κB/p-p65	Yang et al., 2022
AC-YVAD-CMK	ICH mice model	Inhibited pyroptosis	Reducing caspase-1 activation and inhibiting IL-1 β production and maturation	Xu et al., 2020
VX765	CCI mouse model	Inhibited pyroptosis and inflammatory mediator expression	Inhibiting caspase-1 activation and HMGB1/TLR4/NF-kappa B pathway	Dai et al., 2019
VX765	Septic mice model	Reversed cognitive dysfunction	Inhibiting caspase-1	Lin et al., 2018
Sevoflurane	APP/PS1 mice model	Aggravated the progression of AD	Activating NLRP3/caspase-1/GSDMD axis	Sun et al., 2020
PF	Depression mouse model	Alleviated neuroinflammation and exerted antidepressant effects	Inhibiting the enhanced expression of GSDMD and pyroptosis signaling transduction including caspase−1, NLRP3, and IL-1β	Xu et al., 2019b
VX-765	MS animal model, EAE	Reduced pyroptosis	Inhibiting the expression of caspase-1	Wang et al., 2020c
MAF	Mouse BV2 microglia	Inhibited GSDMD cleavage and reduced the levels of inflammatory factors	Directly binding to the GSDMD-Asp275 site	Tian et al., 2021a
LPS and nigericin	APP/PS1 double transgenic mouse model	Improved the memory ability and behavior	Inhibiting the release of inflammatory cytokines	Han et al., 2020b
CD73	C57BL/6J CD73 deficient mice and wild-type mice	Decreased microglia pyroptosis	Suppressing the activation of NLRP3 inflammasome complexes	Xu et al., 2021b

*OGD/R, oxygen-glucose deprivation/reoxygenation; I/R, ischemia-reperfusion; SAH, subarachnoid hemorrhage; TREM-1, triggering receptor expressed on myeloid cells 1; GSDMD-N, N-terminal fragment of GSDMD; IL, interleukin; MCAO, middle cerebral artery occlusion; MVC, maraviroc; ICH, intracerebral hemorrhage; CCR5, C-C chemokine receptor 5; PKA, protein kinase A, CREB, cAMP response element binding; NLRP1, nucleotide-binding domain leucine-rich repeat pyrin domain containing 1; Dex, Dexmedetomidine; p-MCAO, permanent MCAO; P2X7R, purinergic 2X7 receptor; Andro, Andrographolide; SBI, secondary brain injury; TLR4, toll-like receptor 4; NF-κB, nuclear transcription factor-κB; SAFI, Salvianolic Acids for Injection; MCAO/R, MCAO/reperfusion; APP, amyloid precursor protein; PS1, presenilin 1; Rsv, Resveratrol; AlCl3, aluminum chloride; SIRT1, sirtuin 1; KOR, κ opioid receptor; CaMKII, calcium/calmodulin dependent protein kinase II; CREB, cyclic adenosine monophosphate response element binding protein; MPTP, N-methyl-4-phenyl-1,2,3,6-tetrahydropyridine; PD, Parkinson's disease; KAE, kaemperfol; 6-OHDA, 6-hydroxydopamine; Qu, Quercetin; Mn, manganese; PAS-Na, sodium para-aminosalicylic acid; DI, dimethyl itaconate; Nrf 2, nuclear factor erythroid 2 related factor 2; HO 1, heme oxygenase 1; SFN, sulforaphane; DMF, dimethyl fumarate; SCI, spinal cord injury; lncRNAs, long non-coding RNAs; CCI, controlled cortical impact; HMGB1, high-mobility cassette-1; AD, azheimer's disease; PF, paeoniflorin; MAF, mafenide; EAE, experimental autoimmune encephalomyelitis*.

## Summary and Future Prospect

Microglia pyroptosis is now a common cause of secondary neuronal injury. However, direct studies on microglia pyroptosis are still lacking. And there are still many questions related to pyroptosis that remain to be addressed. For example, it is true that some bacteria are thought to have evolved mechanisms to resist pyroptosis and thus evade immunity, such as Shigella. However, it is still widely believed that pathogen-associated PAMP and DAMP activate the pyroptosis pathway by activating GSDMD-associated proteins in the downstream pathway. The pyroptosis pathway has been more extensively studied and more activation pathways have been identified. In addition to the classical caspase-1-mediated pyroptosis pathway, the LPS-mediated caspase-4/5/11 non-classical pyroptosis pathway, and pyroptosis *via* apoptotic transformation.

Currently, inhibition of microglia pyroptosis has been affirmed in various neurological disease models for its associated therapeutic effects. Dexmedetomidine, Andrographolide, Curcumin and Salvianolic acids for injection have shown their relevant therapeutic effects by targeting NLRP3, caspases and GSDMs. This provides new ideas for immunomodulatory therapy of the CNS. However, there are still some questions about the place of NLRP3 in pyroptosis in specific neurological diseases. For example, in a mouse model of OGD studying ischemic stroke, elevated NLRC4 is thought to contribute to microglia pyroptosis without a significant association with NLRP3. Significantly elevated levels of NLRP3 expression in other cells of the striatum of HD mice, but no significant NLRP3 activation was found in microglia. In a CMS-induced depression model in mice, astrocytes in the hippocampus showed cellular pyroptosis, while microglia did not show significant pyroptosis. In contrast, in PD, microglia pyroptosis may be related to α-synuclein concentration. Therefore, the initiation mechanism of microglia pyroptosis in different diseases and its differences in spatial and temporal aspects need to be more elucidated.

## Author Contributions

XW and TW designed this article. TW, XG, and MF wrote the manuscript and prepared the figures. TW, XS, and YD critically revised the manuscript for important intellectual content. All authors read and approved the final manuscript and agreed to be accountable for all aspects of this work.

## Conflict of Interest

The authors declare that the research was conducted in the absence of any commercial or financial relationships that could be construed as a potential conflict of interest.

## Publisher's Note

All claims expressed in this article are solely those of the authors and do not necessarily represent those of their affiliated organizations, or those of the publisher, the editors and the reviewers. Any product that may be evaluated in this article, or claim that may be made by its manufacturer, is not guaranteed or endorsed by the publisher.

## Abbreviations

CNS: central nervous system
NLRP3: NOD-like receptor thermal protein domain associated protein 3
GSDMs: caspase-1 and gasdermins
PAMPs: pathogen-associated molecular patterns
DAMPs: damage-associated molecular patterns
TBI: traumatic brain injury
BBB: blood-brain barrier
LPS: lipopolysaccharide
IFN-γ: interferon-γ
Aβ: amyloid-beta peptides
IL-1β: interleukin-1β
AOPPs: advanced oxidation protein products
SCI: spinal cord injury
ICH: intracerebral hemorrhage
MLKL: mixed lineage kinase domain-like proteins
PRRs: pattern recognition receptors
TLRs: toll-like receptor
AIM2: absent in melanoma 2
MS: multiple sclerosis
PDE8A: phosphodiesterase 8A
cAMP: cyclic adenosine monophosphate
ADP: adenosine diphosphate
MCAO: middle cerebral artery occlusion
NOX4: NADPH oxidase 4
GPX4: glutathione peroxidase 4
AD: Alzheimer's disease
CARD/ASC: caspase activation and recruitment domain
ACEI: angiotensin-converting enzyme I
PD: Parkinson's disease
6-OHDA: 6-hydroxydopamine
MPTP, 1-Methyl-4-phenyl-1, 2, 3: 6-tetrahydropyridine
HD: Huntington's disease
HTT: Huntington's protein
MAPK: mitogen-activated protein kinase
HIF-1α: hypoxia inducible factor-1α
ROS: reactive oxygen species
OGD/R: oxygen-glucose deprivation and reoxygenation
OM-MSCs: olfactory mucosa mesenchymal stem cells
EAE: experimental autoimmune encephalomyelitis
CUMS: chronic unpredictable mild stress
Nrf2: nuclear factor-erythroid 2 -related factor 2
DLB: depressive-like behavior
CSDS: chronic social defeat stress
TREM: triggering receptor expressed on myeloid cells
CCR5: C-C chemokine receptor 5
SAH: subarachnoid hemorrhage
PKA: protein kinase A
CREB: cAMP response element binding
Dex: Dexmedetomidine
P2X7R: purinergic 2X7 receptor
SAFI: salvianolic acids for injection
BIRI: brain ischemia reperfusion injury
CCH: chronic cerebral hypoperfusion
DM: diabetes mellitus
DDX3X: DEAD-box helicase 3 X-linked
SITR1, sirtuin1, CaMKII: Ca2+/calcium/calmodulin-dependent protein kinase II
APP: amyloid precursor protein
PS1: presenilin-1
NF-?B: nuclear factor-kappa B
PAS-NA: Para-aminosalicylic acid
HO-1: heme oxygenase-1
DI: dimethyl itaconate
SFN: sulforaphane
MyD88: myeloid differentiation factor 88
PF: Paeoniflorin
iBMDM: immortalized bone marrow-derived macrophages
MAF: Mafenide
PI3K: phosphatidylinositol 3-kinase.
